# Age-related real-world treatment patterns and outcomes of localised, high-grade osteosarcoma

**DOI:** 10.1016/j.jbo.2026.100767

**Published:** 2026-04-25

**Authors:** Tomohiro Fujiwara, Shintaro Iwata, Akihito Nagano, Kenji Tsuchihashi, Akira Kawai, Toshifumi Ozaki

**Affiliations:** a Department of Orthopaedic Surgery, Okayama University Graduate School of Medicine, Dentistry, and Pharmaceutical Sciences, Okayama, Japan; b Department of Musculoskeletal Oncology, National Cancer Center Hospital, Tokyo, Japan; c Department of Orthopaedic Surgery, Gifu University Graduate School of Medicine, Gifu, Japan; d Department of Medicine and Biosystemic Science, Faculty of Medical Sciences, Kyushu University, Fukuoka, Japan

**Keywords:** Osteosarcoma, Age, Treatment, Outcome

## Abstract

**Introduction:**

There is currently no consensus regarding the standard treatment for osteosarcoma arising in middle-aged and older patients. We aimed to evaluate real-world treatment patterns and oncological outcomes using a nationwide Japanese database and to clarify optimal age-adapted treatment strategies.

**Methods:**

A total of 871 patients with localised, high-grade osteosarcoma registered between 2011 and 2020 in the Japanese Bone and Soft Tissue Tumour Registry were analysed. Treatment patterns and disease-specific survival (DSS) were evaluated according to age groups (≤ 40 years, n = 615; 41–64 years, n = 154; and ≥ 65 years, n = 102).

**Results:**

Trunk tumours were significantly more frequent in patients aged 41–64 (30%) and ≥ 65 years (40%) than in those aged ≤ 40 years (5%). Definitive surgery rates decreased with age (98%, 91%, and 85%, respectively). Perioperative chemotherapy was administered in 92%, 79%, and 34% of patients, with reduced use of methotrexate and cisplatin in patients aged > 40 years. Multivariable analysis identified age ≥ 65 years, absence of surgery, palliative chemotherapy, and adjuvant or palliative radiotherapy as adverse prognostic factors. The 5-year DSS was 79%, 62%, and 45% in patients aged ≤ 40, 41–64, and ≥ 65 years, respectively (*p* < 0.001). DSS was significantly superior in patients undergoing guideline-concordant treatments (wide/radical resection with perioperative chemotherapy) regardless of age. In patients aged > 40 years who underwent guideline-concordant treatments, DSS did not differ between primary chemotherapy and upfront surgery.

**Conclusion:**

Wide/radical resection combined with perioperative chemotherapy remains the mainstay of treatment regardless of age in osteosarcoma management. The optimal sequencing of chemotherapy and surgery warrants a prospective international collaborative study.

## Highlights

• Nationwide registry analyses revealed age-stratified outcomes in localised, high-grade osteosarcoma.
• Wide/radical resection with perioperative chemotherapy remains fundamental irrespective of age.
• Primary chemotherapy and upfront surgery were associated with similar DSS in patients aged > 40 years.
• Optimal timing of chemotherapy and surgery remains uncertain, supporting a prospective age-adapted trial.

## 1 Introduction

Osteosarcoma is a common primary malignant tumour of bone, typically arising in the metaphysis of the long bones near the growth plates, including the distal femur, proximal tibia and proximal humerus [1], [2], [3]. The incidence of osteosarcoma is bimodal, with a first peak in adolescence at around 18 years of age and a second peak in older adults at around 60 years of age [4]. Although most cases of osteosarcoma occur in younger individuals, the proportion and absolute number of middle-aged and elderly patients over 40 years of age have shown an upwards trend in Japan in recent years. According to the Bone Tumour Registry in Japan, 45 of 162 middle-aged patients with osteosarcoma (28%) were registered in 2006, whereas 64 of 177 patients (36%) were registered in 2021, accounting for more than 30% of all cases [5], [6]. Given that populations around the world are rapidly ageing [7], the proportion of osteosarcoma in middle-aged and older patients may also increase in many countries.

More than 85% of patients with osteosarcoma present with localised disease [3]. For localised, high-grade osteosarcoma, the standard treatment for children and young adults aged ≤ 40 years consists of multimodal therapy, including neoadjuvant and adjuvant chemotherapy with methotrexate (MTX), adriamycin (ADR) and cisplatin (CDDP) combined with surgical excision with wide margins [8]. Historically, patients were treated with surgery alone (mainly limb amputation) before the introduction of systemic chemotherapy in the 1970s, but most patients developed distant metastases postoperatively, which resulted in extremely poor outcomes with 2-year disease-free survival rates of less than 15%–20% [9]. The advances in multidisciplinary treatment combining systemic chemotherapy and surgery have improved the 5-year overall survival rate to 60%–70% in patients with localised, high-grade extremity osteosarcoma [10]. Although there is no clear evidence that neoadjuvant (preoperative) chemotherapy improves overall prognosis, neoadjuvant chemotherapy has been widely accepted as a community standard treatment. This would be primarily due to practical reasons, including the fact that the production of expandable endoprostheses for skeletally immature children requires more than 1 month and the potential for tumour shrinkage, which contributes to more functional and safer limb-sparing procedures. However, no consensus exists regarding treatment strategies for middle-aged and older patients aged > 40 years with localised, high-grade osteosarcoma and other histological subtypes of primary malignant bone tumours, including undifferentiated pleomorphic sarcoma of the bone and leiomyosarcoma of the bone.

For patients aged > 40 years, several retrospective studies have been conducted to date. Although the efficacy of adjuvant chemotherapy in this age group remains unknown [9], [11], [12], [13], [14], [15], [16], several retrospective reports from Europe and the USA have indicated the potential benefit of perioperative chemotherapy [9], [11], [14]. Bacci et al. reported a 5-year overall survival rate of 70% in 34 patients aged 41–60 years with localised extremity osteosarcoma using perioperative regimens consisting of ADR, CDDP and ifosfamide (IFO), which was comparable to the outcomes of younger patients [9]. In contrast, Song et al. reported a 5-year metastasis-free survival rate of 40% in 26 patients aged 40–60 years with localised extremity osteosarcoma using MTX, ADR and CDDP, which was lower than the 63.2% observed in patients aged ≤ 40 years [16]. Thus, consensus regarding the optimal treatment for older patients with osteosarcoma has not yet been established. Moreover, nationwide studies investigating real-world treatment patterns are limited and no prospective study has investigated the optimal timing of surgery and perioperative chemotherapy.

Therefore, this study aimed to investigate real-world treatment patterns and outcomes for osteosarcoma using a nationwide database in Japan and to clarify the optimal treatment strategies in accordance with age groups. The following questions were addressed in this study: (1) What are the nationwide real-world trends in osteosarcoma treatment across different age categories (paediatric and young adults, middle-aged and elderly)? and (2) Which treatment strategies are most effective for patients aged 40 years and older?

## 2 Patients and methods

### 2.1 Data source

All data were obtained from the BSTTR, which is a nationwide organ-specific registry for bone and soft-tissue tumours in Japan. The registry was launched in 1950′s and has been organized and funded by the Japanese Orthopaedic Association (JOA) and promoted by National Cancer Centre. Data were collected from 89 JOA-certified hospitals in which registration is mandatory and from other hospitals in which data registration is voluntary. All data, including tumour- and treatment-related data as well as oncological outcomes were registered by the physicians and updated annually. The BSTTR partially corresponds to the SEER database in the United States [17], [18], but has several unique features [19]. Since the BSTTR is an organ-specific cancer registry for bone and soft tissue tumour registered by the treating physicians, several disease-specific detailed data such as the details in histologic findings, treatment modalities, and surgical, functional and oncologic outcomes are collected. On the other hand, the registration does not enumerate whole population and patients treated outside JOA-certified hospitals or by other specialties may not be fully included. However, osteosarcoma is typically managed at specialized orthopedic oncology centers in Japan, except for rare head and neck cases. Accordingly, the registry is considered to capture the vast majority of clinically relevant osteosarcoma cases. Our study was approved by the Institutional Review Board of the Okayama University (IRB No. 2406-019).

### 2.2 Study population

Patients diagnosed with osteosarcoma from 2011 to 2020 were identified from the BSTTR. The inclusion criteria were as follows: patients with a pathologically confirmed osteosarcoma, those with a localised, high-grade disease and for whom the tumour-related information and oncological outcome were available. Patients who were not histologically diagnosed, had been initially treated elsewhere or had missing information on tumour-related information or oncological event were excluded from the study.

### 2.3 Outcome

The primary outcome of this study was the cumulative disease-specific survival (DSS) of all patients in accordance with the age groups (≤ 40 years, 41–64 years and ≥ 65 years) and the secondary outcome was the DSS of patients who underwent surgery of the primary tumour. The data extracted from the BSTTR included basic demographic details (age, sex, status at the first visit (newly diagnosed or referred after initial treatment elsewhere) and date of referral), tumour-related information (date of diagnosis, method of diagnosis (pathology or clinical), tumour grade, tumour site, metastases at the time of diagnosis; treatment-related information (surgery for the primary tumour and for metastases, the use and purpose of chemotherapy or radiotherapy (RT) and the chemotherapeutic drug) and oncological outcome at the final review. Tumours were classified according to the UICC TNM classification system (8th edition) for primary malignant bone tumours. Stage II disease was defined as high-grade tumours without distant metastasis, whereas stage III disease was defined as the presence of skip metastases within the same bone [20]. Surgical margins were recorded using the system established by Enneking et al. [21] as radical, wide, marginal or intralesional.

### 2.4 Statistical analysis

The Kaplan–Meier method was used to determine DSS, with time zero defined as the diagnosis date and censored at the date of the last follow-up. The disease-specific death was determined in each participating institution based on clinician adjudication or on death certificates and then registered in the BSTTR. Univariable analysis was conducted by comparing the groups using the log-rank test and the factors that are biologically considered as significant and without high multicollinearity were incorporated into multivariable analysis. The cause-specific Cox proportional hazard model was used to calculate hazard ratios (HRs) and 95% confidence intervals (CIs). Cancer-related deaths were defined as the event of interest, whereas deaths from other causes and patients alive at last follow-up were treated as censored observations. Correlations between clinicopathological variables and the age group at the time of diagnosis were analysed using the chi-squared or Fisher’s exact test. Statistical comparisons were two-sided and considered significant at *P* < 0.05. All analyses were performed using SPSS (version 23; IBM SPSS, Armonk, NY, USA).

## 3 Results

### 3.1 Patient characteristics and treatment details

A total of 871 patients with localised, high-grade osteosarcoma were studied. Of these patients, 850 (98%) were at stage II at the time of diagnosis and 21 (2%) were at stage III. In addition, 615 (71%), 154 (18%) and 102 (12%) patients were aged ≤ 40 years, 41–64 years and ≥ 65 years, respectively (Table 1). Although no significant difference in tumour size was found among the groups, the proportion of patients with tumours arising in the trunk was significantly higher in patients aged 41–64 years (*n* = 30, 30%) and ≥ 65 years (*n* = 46, 40%) than those aged ≤ 40 years (*n* = 41, 5%; *p* < 0.001). Conventional osteosarcoma accounted for more than 70% of cases across all age groups. Dedifferentiated parosteal osteosarcoma was more frequently observed in middle-aged and older patients, while secondary osteosarcoma was most common in patients aged ≥ 65 years (Supplementary Table 1).

**Table 1:** Patient characteristics.

	Overall		≤ 40 years		41–64 years		≥ 65 years		*p* value
	N	%	N	%	N	%	N	%	
Total	871		615	71%	154	18%	102	12%	
Sex									0.217
Male	497	57%	357	58%	90	58%	50	49%	
Female	374	43%	258	42%	64	42%	52	51%	
Site									<0.001
Limb	754	87%	585	95%	108	70%	61	60%	
Femur	434	50%	319	52%	75	49%	40	39%	
Tibia	192	22%	155	25%	24	16%	13	13%	
Humerus	75	9%	68	11%	5	3%	2	2%	
Others	53	6%	43	7%	4	3%	6	6%	
Trunk	117	13%	30	5%	46	30%	41	40%	
Pelvis	60	7%	14	2%	28	18%	18	18%	
Others	57	7%	16	3%	18	12%	23	23%	
Size									0.138
≤ 8 cm	421	48%	286	47%	77	50%	58	57%	
> 8 cm	450	52%	329	53%	77	50%	44	43%	
Surgery of primary tumour									<0.001
Yes	830	95%	603	98%	140	91%	87	85%	
No	41	5%	12	2%	14	9%	15	15%	
Resection margin (surgical cases)									<0.001
Wide	772	94%	574	96%	122	88%	76	88%	
Marginal	25	3%	15	3%	8	6%	2	2%	
Intralesional	21	3%	7	1%	6	4%	8	9%	
Radical	5	1%	3	1%	2	1%	0	0%	
Unknown	7	1%	4	1%	2	1%	1	1%	
Chemotherapy									<0.001
Yes	788	90%	606	99%	138	90%	44	43%	
No	83	10%	9	1%	16	10%	58	57%	
Chemotherapy purpose									0.003
Adjuvant	719	83%	563	92%	121	79%	35	34%	
Palliative	69	8%	43	7%	17	11%	9	9%	
Chemotherapy sequence (adjuvant setting)									<0.001
Preop. + postop.	599	83%	506	90%	78	57%	15	43%	
Preop.	64	9%	35	6%	20	14%	9	26%	
Postop.	56	8%	22	4%	23	17%	11	31%	
Chemotherapeutic agents (adjuvant setting)									
MTX	565	79%	512	91%	50	50%	3	9%	<0.001
ADR	686	95%	537	95%	118	98%	31	89%	0.083
CDDP	638	89%	531	94%	92	76%	15	43%	<0.001
IFO	431	60%	328	58%	84	69%	19	54%	0.059
Radiotherapy									<0.001
Yes	126	14%	51	8%	35	23%	40	39%	
No	745	86%	564	92%	119	77%	62	61%	
Radiotherapy purpose									0.862
Radical	58	7%	21	3%	16	10%	21	21%	
Adjuvant	16	2%	7	1%	4	3%	5	5%	
Palliative	52	6%	23	4%	15	10%	14	14%	
Radiotherapy sequence (adjuvant setting)									0.752
Preop.	2	0.2%	1	0.2%	0	0%	1	1%	
Postop.	12	1%	5	1%	3	2%	4	4%	
Intraop.	2	0.2%	1	0.2%	1	1%	0	0%	

Abbreviations; MTX, methotrexate; ADR, adriamycin; CDDP, cisplatin; IFO, ifosfamide.

Treatment patterns also varied in accordance with the age groups. The proportion of patients who underwent definitive surgery decreased with age, being 98%, 91% and 85% in patients aged ≤ 40, 41–64 and ≥ 65 years, respectively (*p* < 0.001). The use of chemotherapy similarly declined with age (99%, 90% and 43% in patients aged ≤ 40, 41–64 and ≥ 65 years, respectively; *p* < 0.001). Perioperative chemotherapy was administered in 92%, 79% and 34% of these age groups (*p* = 0.003), indicating that a substantial proportion of patients aged 41–64 years still received perioperative chemotherapy.

In patients aged ≤ 40, 41–64 and ≥ 65 years who received perioperative chemotherapy, 90%, 57% and 43% received pre- and post-operative chemotherapy; 6%, 14% and 26% received preoperative chemotherapy only and 4%, 17% and 31% received post-operative chemotherapy only, respectively. Thus, preoperative chemotherapy was administered in 96%, 71% and 69% of these groups, indicating that most patients aged 41–64 years received perioperative chemotherapy, similar to younger patients.

In terms of chemotherapeutic drug, MTX and CDDP were used less frequently in patients aged > 40 years who received perioperative chemotherapy. MTX was administered to 91%, 50% and 9% of patients aged ≤ 40, 41–64 and ≥ 65 years, respectively (*p* < 0.001) and CDDP was administered to 94%, 76% and 43% of these patients (*p* < 0.001). Conversely, the use of RT increased with age (8%, 23% and 39%; *p* < 0.001), which may be related to the higher proportion of trunk tumours and the greater difficulty in achieving a wide resection margin in these patients. Furthermore, the proportion of intralesional margins increased with age (1%, 4% and 9%; *p* < 0.001).

### 3.2 Survival outcomes and prognostic factors according to age groups

**Fig. 1:**
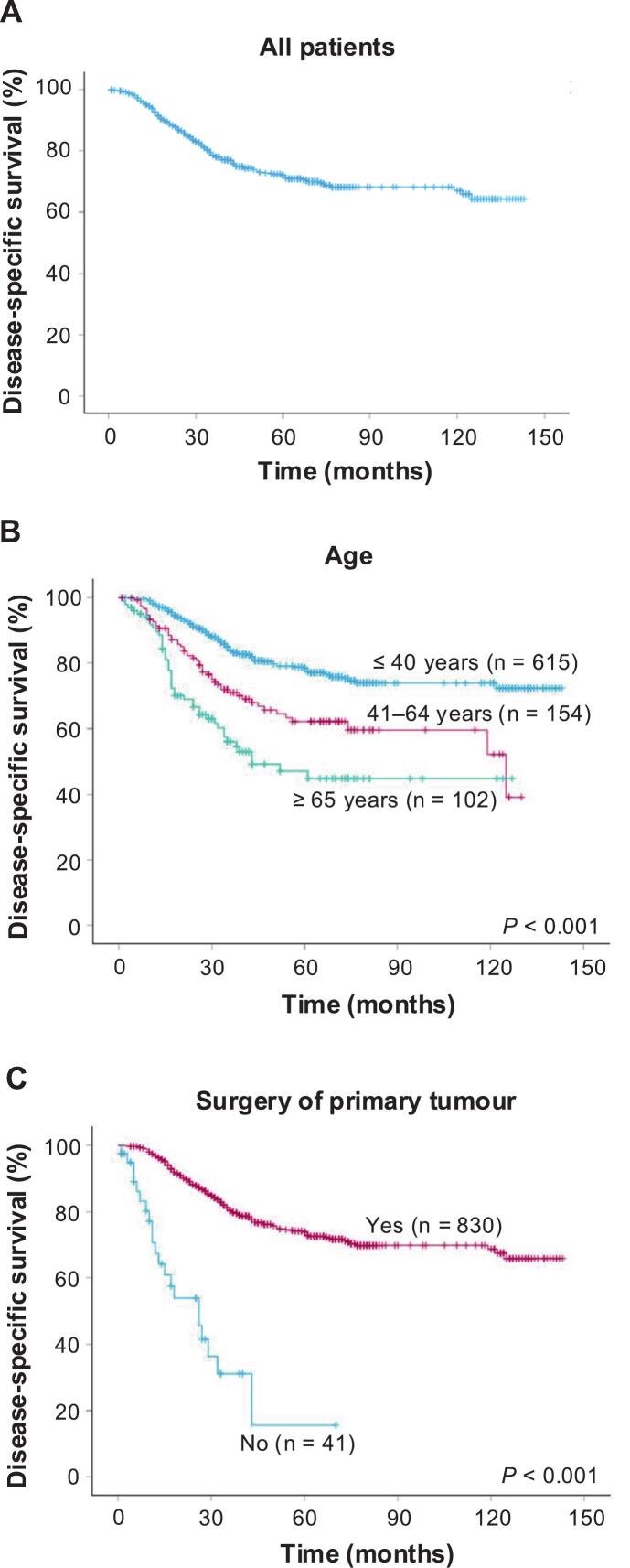
Kaplan–Meier curves showing disease-specific survival for all patients (A), patients stratified by age group (B) and patients stratified by whether surgery of the primary tumour was performed (C).

The 5-year and 10-year DSS for all patients were 72% and 67%, respectively (Fig. 1A). When stratified by age group, the 5-year DSS was 79%, 62% and 45% in patients aged ≤ 40, 41–64 and ≥ 65 years, respectively (Fig. 1B; *p* < 0.001). Among the tumour-related factors, surgery of the primary tumour significantly stratified the DSS; the 5-year DSS was 74% and 16% in patients with and without surgical treatment, respectively (Fig. 1C; *p* < 0.001). Multivariable analyses revealed that age ≥ 65 years (HR = 2.16; 95%CI, 1.37–3.42 versus ≤ 40 years, reference, *p* < 0.001), no surgery of the primary tumour (HR = 1.92; 95%CI, 1.12–3.28 versus surgery of the primary tumour, reference, *p* = 0.018), palliative chemotherapy (HR = 3.34; 95%CI, 1.88–5.92 versus no chemotherapy, reference, *p* < 0.001), adjuvant RT (HR = 1.74; 95%CI, 1.09–2.78 versus no RT, reference, *p* = 0.020) and palliative RT (HR = 3.41; 95%CI, 2.26–5.14 versus no RT, reference, *p* < 0.001) were significantly associated with worse DDS (Table 2).

**Table 2:** Multivariable analyses for disease-specific survival.

	N	HR	95% CI	*p* value
Total	871			
Age				
≤ 40 years	615		Reference	
41–64 years	154	1.41	0.98–2.02	0.067
≥ 65 years	102	2.16	1.37–3.42	<0.001
Site				
Lower limb	664		Reference	
Upper limb	90	0.83	0.49–1.42	0.497
Trunk	117	1.47	0.99–2.20	0.058
Size				
≤ 8 cm	421		Reference	
> 8 cm	450	1.16	0.88–1.52	0.287
Surgery of primary tumour				
Yes	830		Reference	
No	41	1.92	1.12–3.28	0.018
Chemotherapy purpose				
No	83		Reference	
Adjuvant	719	1.11	0.67–1.86	0.683
Palliative	69	3.34	1.88–5.92	<0.001
Radiotherapy purpose				
No	745		Reference	
Radical	16	2.05	0.94–4.46	0.072
Adjuvant	58	1.74	1.09–2.78	0.020
Palliative	52	3.41	2.26–5.14	<0.001

Multivariable analyses were also performed in accordance with age groups. Trunk tumour (HR = 2.45; 95%CI, 1.32–4.58 versus lower extremity, reference, *p* = 0.005) and palliative RT (HR = 9.90; 95%CI, 3.38–9.90 versus no RT, reference, *p* < 0.001) in patients aged ≤ 40 years, tumour larger than 8 cm (HR = 2.08; 95%CI, 1.15–3.79 versus tumour ≤ 8 cm, reference, *p* = 0.016) and palliative RT (HR = 5.29; 95%CI, 2.38–11.78 versus no RT, reference, *p* < 0.001) in patients aged 41–65 years and no surgery of the primary tumour (HR = 3.02; 95%CI, 1.03–8.87 versus no surgery, reference, *p* = 0.045), palliative chemotherapy (HR = 3.51; 95%CI, 1.28–9.63 versus no chemotherapy, reference, *p* = 0.015) and radical RT (HR = 3.42; 95%CI, 1.09–10.72 versus no RT, reference, *p* = 0.035) in patients aged > 65 years were significantly associated with worse DSS (Supplementary Table 2).

### 3.3 Subgroup analyses in patients who underwent definitive surgery of the primary tumour

**Fig. 2:**
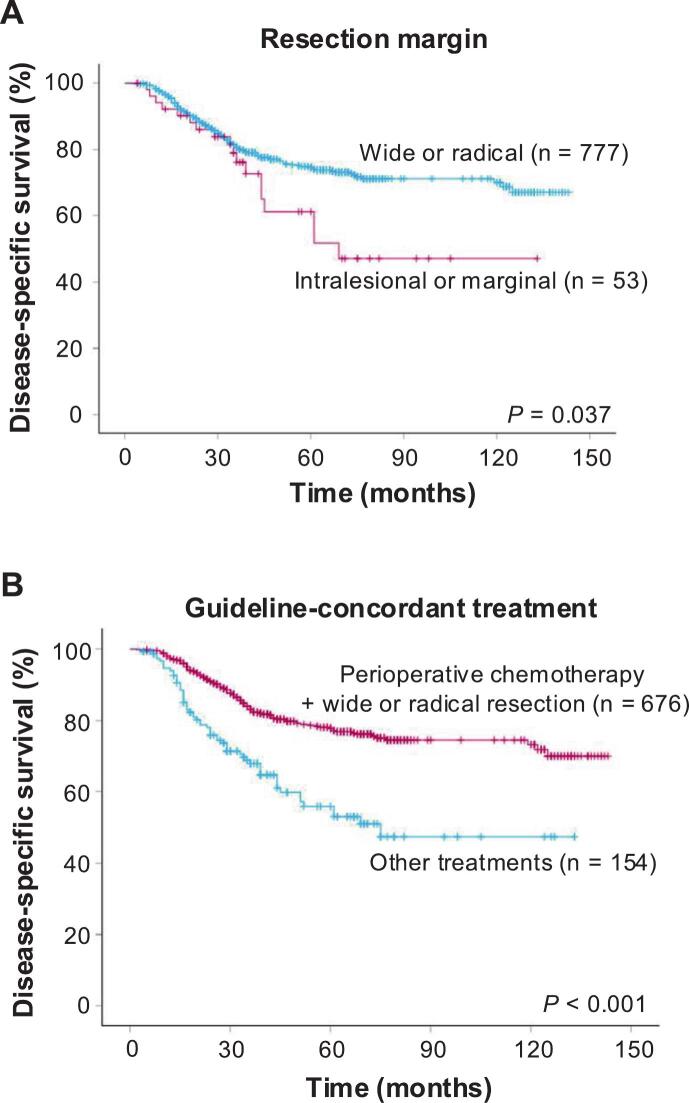
Kaplan–Meier curves showing disease-specific survival in patients who underwent surgery of the primary tumour, stratified by surgical margin status (A) and guideline-concordant treatment (B).

In patients who underwent definitive surgery of the primary tumour, the margin status was significantly associated with DSS; the 5-year DSS of patients with intralesional or marginal margins was 61%, which was significantly poorer than those with wide or radical margins (5-year DSS, 75%; *p* = 0.037; Fig. 2A). Notably, treatments that were concordant to the clinical practice guidelines (CPGs), which consist of wide or radical resection of the tumour and perioperative chemotherapy, significantly stratified DSS; the 5-year DSS was 78% and 56% in patients with and without guideline-concordant treatments, respectively (*p* < 0.001; Fig. 2B). In multivariable analyses, trunk tumour (HR = 1.72; 95%CI, 1.15–2.56 versus lower extremity, reference, *p* = 0.008), treatments that were not concordant to CPGs (HR = 1.54; 95%CI, 1.09–2.18 versus guideline-concordant treatments, reference, *p* = 0.016) and palliative RT (HR = 4.98; 95%CI, 3.36–7.38 versus no RT, reference, *p* < 0.001) were significantly associated with worse DSS (Supplementary Table 3).

### 3.4 Subgroup analyses in patients who underwent guideline-concordant treatments

**Fig. 3:**
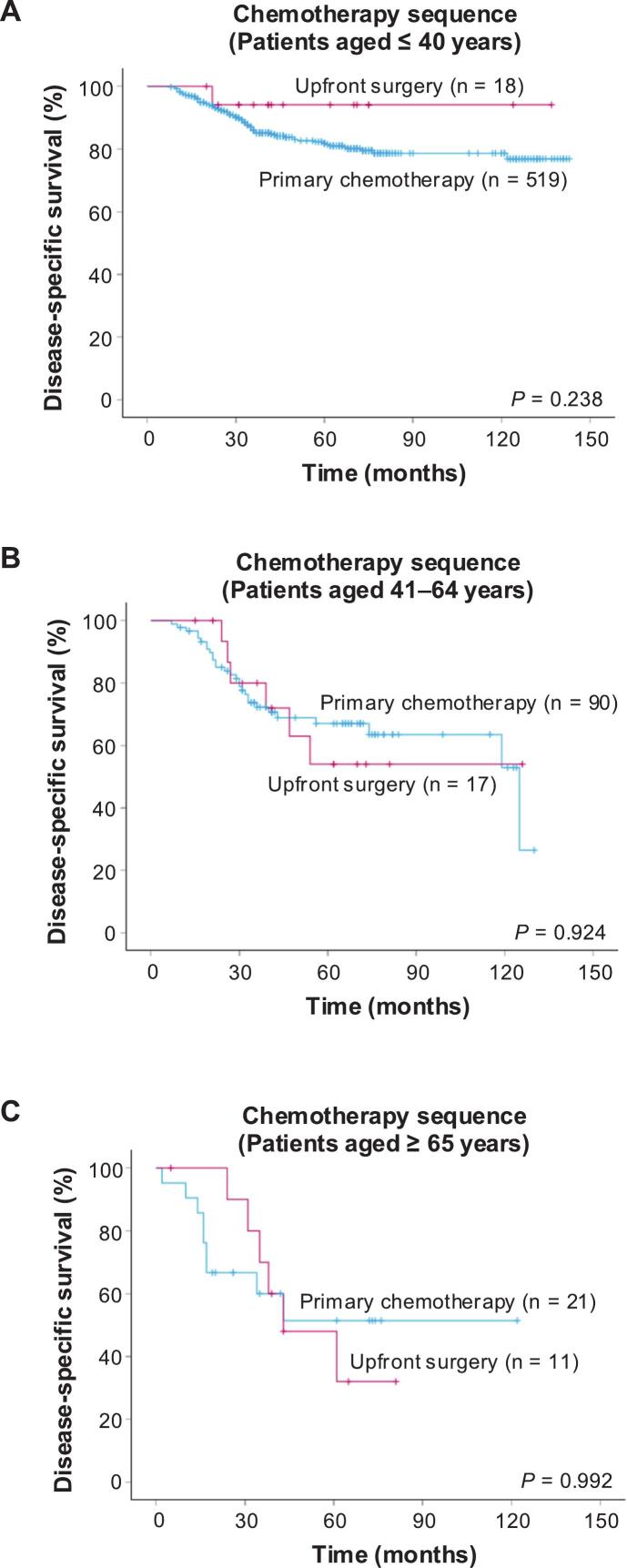
Kaplan–Meier curves showing disease-specific survival in patients who underwent wide or radical resection combined with perioperative chemotherapy for patients aged ≤ 40 years (A), patients aged 41–64 years (B) and patients aged ≥ 65 years (C).

In patients who underwent wide or radical resection and adjuvant chemotherapy, the survival outcomes were compared in accordance with the timing of surgical treatment. Comparing the DSS of patients who underwent primary chemotherapy with that of patients who underwent upfront surgery, no significant difference was found between the two groups (*p* = 0.200; Supplementary Fig. 1A). In patients aged ≤ 40 years, although the difference was not statistically significant, the upfront surgery group tended to show better DSS (*p* = 0.238; Fig. 3A). In patients aged > 40 years, the DSS of those who underwent upfront surgery was comparable to that of patients who received primary chemotherapy, with no statistically significant difference (*p* = 0.629; Supplementary Fig. 1B). These findings were consistent in subgroup analyses of patients aged 41–65 years (*p* = 0.924; Fig. 3B) and ≥ 65 years (*p* = 0.992; Fig. 3C). In multivariate analyses, age 41–65 years (HR = 1.56; 95%CI, 1.02–2.39 versus ≤ 40 years, reference, *p* = 0.042), age ≥ 65 years (HR = 1.97; 95%CI, 1.10–3.52 versus ≤ 40 years, reference, *p* = 0.023), trunk tumour (HR = 2.24; 95%CI, 1.41–3.56 versus lower extremity, reference, *p* < 0.001) and palliative RT (HR = 6.15; 95%CI, 3.79–9.99 versus no RT, reference, *p* < 0.001) were significantly associated with worse DSS (Supplementary Table 4).

## 4 Discussion

Although there is currently no consensus regarding the standard treatment for patients aged > 40 years with localised, high-grade osteosarcoma, nationwide real-world data on treatment patterns and outcomes have not been well documented. This nationwide analysis confirmed that perioperative chemotherapy was administered in a substantial proportion of patients aged > 40 years. Perioperative chemotherapy was delivered in 92%, 79% and 34% of patients aged ≤ 40, 41–64 and ≥ 65 years, respectively, which was comparable to or slightly higher than previously reported rates in patients aged > 40 years [9], [11], [14]. In a retrospective study of 53 patients aged 40–60 years, Bacci et al. reported that perioperative chemotherapy combined with surgery was performed in 55% of cases, whereas 45% underwent surgery alone [9]. In an EMSOS study of 220 patients aged > 40 years, Grimer et al. reported that perioperative chemotherapy combined with surgery was performed in 57% of cases, whereas 27% were treated with surgery alone [11]. Similarly, Manoso et al. reported that perioperative chemotherapy combined with surgery was performed in 69% of 58 patients aged > 40 years, with 31% undergoing surgery alone [14]. With regard to chemotherapy sequence, neoadjuvant chemotherapy was performed in 63% and 69% of cases who underwent perioperative chemotherapy in the aforementioned previous studies conducted by Grimer [11] and Manoso [14], respectively. In the present study, in patients aged 41–64 and ≥ 65 years who received adjuvant chemotherapy, 71% and 69% commenced treatment with neoadjuvant therapy, respectively. These findings indicate that neoadjuvant chemotherapy is widely administered to patients aged > 40 years worldwide, which is similar to that used in younger patients.

The present study confirmed the clear stratification of the survival outcomes in accordance with the age groups at diagnosis in patients with localised, high-grade osteosarcoma. The 5-year DSS was 79% and 62% in patients aged ≤ 40 and 41–65 years, respectively, but only 45% in those aged ≥ 65 years, indicating worse prognosis in the elderly cohort; multivariable analysis confirmed that age ≥ 65 years is an independent adverse prognostic factor. This result was consistent with the findings of Tsuda et al., who demonstrated that advanced age was an unfavourable prognostic factor in a cohort including metastatic disease at diagnosis [22]. The present study, which focused on a localised, high-grade disease, similarly showed a significantly worse prognosis in elderly patients. This finding would be attributed to several factors. First, the proportion of trunk tumour was significantly higher in elderly patients, which are associated with the indication of surgical excision of the primary tumour. Second, the proportion of secondary osteosarcoma increased with age. Of note, post-radiation osteosarcoma, is associated with worse outcomes than conventional osteosarcoma [23], which may have contributed to the inferior survival observed in older patients. Third, key drugs such as MTX and CDDP could not be administered in a substantial proportion of cases. Hayakawa et al. reported nephrotoxicity in 5 of 19 patients (26%) aged > 40 years who were treated with CDDP, whereas no nephrotoxicity related to IFO was observed [6]. Therefore, a sufficient dose intensity could not be maintained even when these drugs were administered. Fourth, emerging molecular evidence indicates that osteosarcoma occurring in middle-aged to elderly patients may have distinct biological characteristics, which may reduce chemosensitivity compared with younger patients. Recent genomic analyses have revealed that osteosarcoma in elderly patients frequently harbours specific genetic alterations such as *TP53*, *LSAMP* and *H3F3A* mutations and exhibits a distinct DNA methylation profile [24]. More recently, Otani et al. reported that alterations in *CCNE1*, *MCL1*, *MYC* and *RB1* were significantly associated with younger age, whereas alterations in *CDK4*, *CDKN2A*, *CDKN2B*, *H3F3A*, *KMT2D*, *MDM2*, *RAC1* and *SETD2* were significantly associated with older age, indicating that mutational signatures correlated with age may influence chemosensitivity [25]. These findings reinforce the hypothesis that tumour biology in the elderly differs substantially from that in younger patients. Collectively, these findings highlight the importance of developing novel treatment strategies for middle-aged patients, including effective standardised chemotherapy regimens or novel therapeutic drugs that are feasible and safe for these patients. Of note, the association between palliative chemotherapy or RT and worse survival likely reflects selection bias, as these treatments are preferentially administered to patients with more advanced or unresectable disease, rather than indicating a detrimental effect of these modalities.

To date, no consensus exists regarding the optimal treatment strategy for osteosarcoma in middle-aged and elderly patients. In the present study, favourable outcomes were observed in patients who underwent wide resection combined with perioperative chemotherapy regardless of age; therefore, perioperative chemotherapy should be recommended for middle-aged patients who are tolerable to systemic treatments. However, the optimal timing of perioperative chemotherapy remains unclear. In our cohort, neoadjuvant and adjuvant chemotherapy combined with surgery versus upfront surgery followed by adjuvant chemotherapy achieved comparable outcomes. Furthermore, a trend towards better DSS was observed in the upfront surgery group especially in younger patients. Our findings were consistent with the results of a randomised trial for paediatric patients published in 2003 (POG-8651). A total of 106 paediatric patients aged ≤ 30 years with nonmetastatic osteosarcoma were randomly assigned to immediate surgery or presurgical chemotherapy, which showed a trend towards improved event-free survival (5-year, 69% versus 61%) and overall survival (5-year, 79% versus 76%) in the immediate surgery group compared with the primary chemotherapy group [26]. Similarly, a recent retrospective study using the SEER database showed a trend towards better overall survival in patients who underwent upfront surgery; the 5-year OS was 74% and 67% in patients with upfront surgery and primary chemotherapy, respectively. Therefore, Danese et al. suggested to revisit a prospective trial of osteosarcoma treatment regarding the timing of surgery and chemotherapy [27]. We consider that middle-aged patients, whose response to chemotherapy is poorer than that of younger patients, would be an appropriate population for these trials.

The only prospective investigation focusing on osteosarcoma in middle-aged and elderly patients has been the EURO-B.O.S.S. study, which is an observational, registry-based study. Ferrari et al. reported the outcomes of this study in 2017[28]. Among 151 localised cases, 110 (73%) received primary chemotherapy, in which good responder (necrosis rate ≥ 90%) was only 22 (21%) and a trend towards superior prognosis was observed in the upfront surgery group; the 5-year disease-free survival was 48% in the pre- and post-operative chemotherapy group and 64% in the post-operative chemotherapy group (*p* = 0.2) [28]. Similar findings were recently reported from a retrospective study conducted by Hayakawa et al. [6]. In the Cancer Institute Hospital in Tokyo, the treatment protocol was basically neoadjuvant chemotherapy with three courses followed by surgery and adjuvant chemotherapy before 2006, whereas surgery of the primary tumour was prioritised after 2006. Consequently, a trend towards improved survival outcomes was observed after 2006; the 5-year survival rate was 57.9% before 2006 and 78.8% after 2006 (*p* = 0.1958) [6]. Considering these retrospective data, a prospective, randomised trial is warranted to validate these findings. Furthermore, considering that the incidence of osteosarcoma in this age group is limited, an international collaboration will be essential to establish the standard treatment strategy for osteosarcoma in patients aged > 40 years.

This study has several limitations. First, the BSTTR database does not include the exact doses and toxicity of chemotherapy and RT. Thus, the efficacy and safety of these therapies could not be precisely evaluated on the basis of the dose of administration. Second, there might be a potential for errors in the registration process, as observed in other retrospective studies. Third, our results may not be fully generalisable. Some patients who received care at non-JOA-certified hospitals might not be registered in the database because the registry is not mandatory for these institutions. Fourth, the possibility of a duplicate registration may not be excluded if a patient received care at more than one hospital. However, the BSTTR is designed to automatically exclude the cases if they were referred for second opinion or only observation after treatment in the previous hospital, to avoid duplicate reporting. Despite these limitations, this study provides valuable data, presenting the national trend and outcomes in the era of modern multidisciplinary treatments.

In summary, our nationwide analysis confirmed that advanced age is associated with poor prognosis in localised, high-grade osteosarcoma. Nevertheless, wide resection combined with perioperative chemotherapy, which is equivalent to guideline-concordant treatments, remains the fundamental principle of treatment strategy providing chances for cure regardless of age. We confirmed that perioperative chemotherapy was administered in a substantial proportion of patients aged over 40 years. However, no survival advantage of neoadjuvant and adjuvant chemotherapy over adjuvant chemotherapy was found in our cohort. Considering the poor response to chemotherapy generally observed in middle-aged and older patients, upfront surgery followed by adjuvant chemotherapy may represent a reasonable strategy in this age group. A prospective trial with international collaborations is warranted to validate these findings and to establish age-adapted treatment algorithms for patients aged > 40 years.

## CRediT authorship contribution statement

**Tomohiro Fujiwara:** Writing – original draft, Methodology, Investigation, Funding acquisition, Formal analysis, Data curation, Conceptualization. **Shintaro Iwata:** Writing – review & editing, Validation, Supervision, Project administration. **Akihito Nagano:** Writing – review & editing, Validation. **Kenji Tsuchihashi:** Writing – review & editing, Validation. **Akira Kawai:** Writing – review & editing, Resources. **Toshifumi Ozaki:** Writing – review & editing, Supervision, Resources.

## Declaration of competing interest

The authors declare that they have no known competing financial interests or personal relationships that could have appeared to influence the work reported in this paper.

## Appendix A Supplementary data

Supplementary Data 1 Supplementary data include Kaplan–Meier analyses of disease-specific survival according to chemotherapy sequence in patients who underwent guideline-concordant treatments, and multivariable analyses for disease-specific survival stratified by age group, surgical treatment, and guideline-concordant treatment (Supplementary Tables 1–3).

## References

[1] Link M.P., Goorin A.M., Miser A.W., Green A.A., Pratt C.B., Belasco J.B., Pritchard J., Malpas J.S., Baker A.R., Kirkpatrick J.A. (1986). The effect of adjuvant chemotherapy on relapse-free survival in patients with osteosarcoma of the extremity. N. Engl. J. Med..

[2] Whelan J.S., Davis L.E. (2018). Osteosarcoma, chondrosarcoma, and chordoma. J. Clin. Oncol..

[3] Gill J., Gorlick R. (2021). Advancing therapy for osteosarcoma. Nat. Rev. Clin. Oncol..

[4] Beird H.C., Bielack S.S., Flanagan A.M., Gill J., Heymann D., Janeway K.A., Livingston J.A., Roberts R.D., Strauss S.J., Gorlick R. (2022). Osteosarcoma. Nat. Rev. Dis. Primers.

[5] Committee J.O.A.M.T. (2021).

[6] Hayakawa K., Matsumoto S., Ae K., Tanizawa T., Funauchi Y., Minami Y., Saito M., Okawa A. (2020). Definitive surgery of primary lesion should be prioritized over preoperative chemotherapy to treat high-grade osteosarcoma in patients aged 41-65 years. J. Orthop. Traumatol..

[7] Beard J.R., Officer A., de Carvalho I.A., Sadana R., Pot A.M., Michel J.P., Lloyd-Sherlock P., Epping-Jordan J.E., Peeters G., Mahanani W.R., Thiyagarajan J.A., Chatterji S. (2016). The World report on ageing and health: a policy framework for healthy ageing. Lancet.

[8] P.G. Casali, S. Bielack, N. Abecassis, H.T. Aro, S. Bauer, R. Biagini, S. Bonvalot, I. Boukovinas, J. Bovee, B. Brennan, T. Brodowicz, J.M. Broto, L. Brugieres, A. Buonadonna, E. De Alava, A.P. Dei Tos, X.G. Del Muro, P. Dileo, C. Dhooge, M. Eriksson, F. Fagioli, A. Fedenko, V. Ferraresi, A. Ferrari, S. Ferrari, A.M. Frezza, N. Gaspar, S. Gasperoni, H. Gelderblom, T. Gil, G. Grignani, A. Gronchi, R.L. Haas, B. Hassan, S. Hecker-Nolting, P. Hohenberger, R. Issels, H. Joensuu, R.L. Jones, I. Judson, P. Jutte, S. Kaal, L. Kager, B. Kasper, K. Kopeckova, D.A. Krakorova, R. Ladenstein, A. Le Cesne, I. Lugowska, O. Merimsky, M. Montemurro, B. Morland, M.A. Pantaleo, R. Piana, P. Picci, S. Piperno-Neumann, A.L. Pousa, P. Reichardt, M.H. Robinson, P. Rutkowski, A.A. Safwat, P. Schoffski, S. Sleijfer, S. Stacchiotti, S.J. Strauss, K. Sundby Hall, M. Unk, F. Van Coevorden, W.T.A. van der Graaf, J. Whelan, E. Wardelmann, O. Zaikova, J.Y. Blay, P. Esmo Guidelines Committee, E. Ern, Bone sarcomas: ESMO-PaedCan-EURACAN Clinical Practice Guidelines for diagnosis, treatment and follow-up, Annals of oncology : official journal of the European Society for Medical Oncology / ESMO 29(Suppl 4) (2018) iv79–iv95. 10.1093/annonc/mdy310 30285218

[9] Bacci G., Ferrari S., Mercuri M., Longhi A., Fabbri N., Galletti S., Forni C., Balladelli A., Serra M., Picci P. (2007). Neoadjuvant chemotherapy for osteosarcoma of the extremities in patients aged 41–60 years: outcome in 34 cases treated with adriamycin, cisplatinum and ifosfamide between 1984 and 1999. Acta Orthop..

[10] Goorin A.M., Schwartzentruber D.J., Devidas M., Gebhardt M.C., Ayala A.G., Harris M.B., Helman L.J., Grier H.E., Link M.P. (2003). Presurgical chemotherapy compared with immediate surgery and adjuvant chemotherapy for nonmetastatic osteosarcoma: Pediatric Oncology Group Study POG-8651. J. Clin. Oncol..

[11] Grimer R., Cannon S., Taminiau A., Bielack S., Kempf-Bielack B., Windhager R., Dominkus M., Saeter G., Bauer H., Meller I. (2003). Osteosarcoma over the age of forty. Eur. J. Cancer.

[12] Iwata S., Ishii T., Kawai A., Hiruma T., Yonemoto T., Kamoda H., Asano N., Takeyama M. (2014). Prognostic factors in elderly osteosarcoma patients: a multi-institutional retrospective study of 86 cases. Ann. Surg. Oncol..

[13] Joo M.W., Shin S.H., Kang Y.K., Kawai A., Kim H.S., Asavamongkolkul A., Jeon D.G., Kim J.D., Niu X., Tsuchiya H., Puri A., Wang E.H., Chung S.H., Chung Y.G. (2015). Osteosarcoma in Asian populations over the age of 40 years: a multicenter study. Ann. Surg. Oncol..

[14] Manoso M.W., Healey J.H., Boland P.J., Athanasian E.A., Maki R.G., Huvos A.G., Morris C.D. (2005). De novo osteogenic sarcoma in patients older than forty: benefit of multimodality therapy. Clinical Orthopaedics and Related Research®.

[15] Nagano A., Ishimaru D., Nishimoto Y., Akiyama H., Kawai A. (2017). Primary bone sarcomas in patients over 40 years of age: a retrospective study using data from the Bone Tumor Registry of Japan. J. Orthop. Sci..

[16] Song W., Kong C.-B., Jeon D.-G., Cho W., Kim M., Lee J., Yoo J., Kim J., Lee S.-Y. (2010). Prognosis of extremity osteosarcoma in patients aged 40–60 years: a cohort/case controlled study at a single institute. European Journal of Surgical Oncology (EJSO).

[17] James B.Y.M., Gross C.P., Wilson L.D., Smith B.D. (2009). NCI SEER public-use data: applications and limitations in oncology research. Oncology.

[18] Warren J.L., Klabunde C.N., Schrag D., Bach P.B., Riley G.F. (2002). Overview of the SEER-Medicare data: content, research applications, and generalizability to the United States elderly population. Med. Care.

[19] Ogura K., Higashi T., Kawai A. (2017). Statistics of bone sarcoma in Japan: Report from the Bone and Soft Tissue Tumor Registry in Japan. J. Orthop. Sci..

[20] Brierley J.D., Gospodarowicz M.K., Wittekind C. (2017). TNM Classification of Malignant Tumours.

[21] Enneking W.F., Spanier S.S., Goodman M.A. (1980). A system for the surgical staging of musculoskeletal sarcoma. Clin. Orthop. Relat. Res..

[22] Tsuda Y., Ogura K., Shinoda Y., Kobayashi H., Tanaka S., Kawai A. (2018). The outcomes and prognostic factors in patients with osteosarcoma according to age: a Japanese nationwide study with focusing on the age differences. BMC Cancer.

[23] Sheppard D.G., Libshitz H.I. (2001). Post-radiation sarcomas: a review of the clinical and imaging features in 63 cases. Clin. Radiol..

[24] Koelsche C., Schrimpf D., Tharun L., Roth E., Sturm D., Jones D.T., Renker E.-K., Sill M., Baude A., Sahm F. (2017). Histone 3.3 hotspot mutations in conventional osteosarcomas: a comprehensive clinical and molecular characterization of six H3F3A mutated cases. Clinical Sarcoma Research.

[25] Outani H., Ikegami M., Imura Y., Nakai S., Takami H., Kotani Y., Inoue A., Okada S. (2025). Age-related genomic alterations and chemotherapy sensitivity in osteosarcoma: insights from cancer genome profiling analyses. Int. J. Clin. Oncol..

[26] Goorin A.M., Schwartzentruber D.J., Devidas M., Gebhardt M.C., Ayala A.G., Harris M.B., Helman L.J., Grier H.E., Link M.P., G. Pediatric Oncology (2003). Presurgical chemotherapy compared with immediate surgery and adjuvant chemotherapy for nonmetastatic osteosarcoma: Pediatric Oncology Group Study POG-8651. J. Clin. Oncol..

[27] Danese M.D., Groundland J.S. (2025). Effect of chemotherapy and surgery timing on mortality in upper and lower extremity osteosarcoma. JNCI: J. Natl. Cancer Inst..

[28] Ferrari S., Bielack S.S., Smeland S., Longhi A., Egerer G., Sundby Hall K., Donati D., Kevric M., Brosjo O., Comandone A., Werner M., Monge O., Palmerini E., Berdel W.E., Bjerkehagen B., Paioli A., Lorenzen S., Eriksson M., Gambarotti M., Tunn P.U., Jebsen N.L., Cesari M., von Kalle T., Ferraresi V., Schwarz R., Bertulli R., Kasparek A.K., Grignani G., Krasniqi F., Sorg B., Hecker-Nolting S., Picci P., Reichardt P., Euro-B.O.S.S. (2018). A European study on chemotherapy in bone-sarcoma patients aged over 40: Outcome in primary high-grade osteosarcoma. Tumori.

