# Supplementary material for: Age-related real-world treatment patterns and outcomes of localised, high-grade osteosarcoma
Source: J Bone Oncol. 2026 Apr 25;58:100767. doi: 10.1016/j.jbo.2026.100767 (PMC13156681; doi:10.1016/j.jbo.2026.100767)
Supplement: Supplementary Data 1 — Supplementary data include Kaplan–Meier analyses of disease-specific survival according to chemotherapy sequence in patients who underwent guideline-concordant treatments, and multivariable analyses for disease-specific survival stratified by age group, surgical treatment, and guideline-concordant treatment (Supplementary Tables 1–3). [file mmc1.pdf]

Supplementary Figure 1

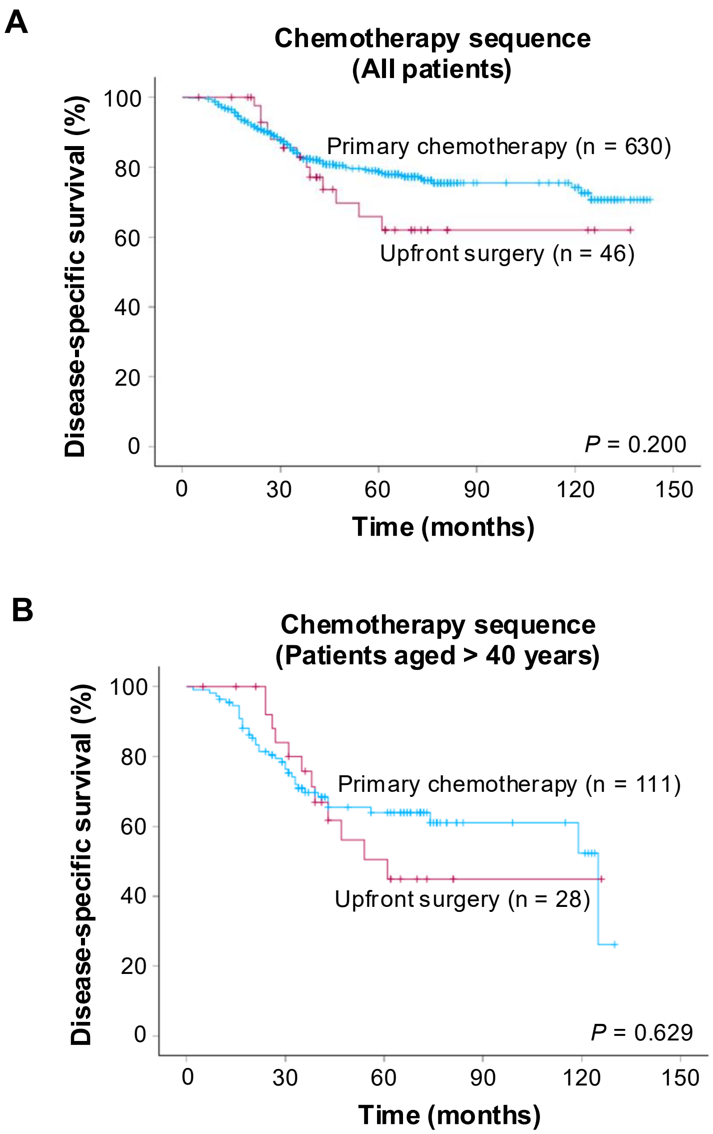

Supplementary Figure 1. Kaplan–Meier curves showing disease-specific survival in patients who underwent wide or radical resection combined with perioperative chemotherapy for all patients (A) and patients aged > 40 years (B).

Supplementary Table 1. Distribution of histologic subtypes in accordance with age groups

	Overall		≤ 40 years		41–64 years		≥ 65 years	
	N	%	N	%	N	%	N	%
Conventional	648	74%	463	75%	108	70%	77	75%
Telangiectatic	21	2%	16	3%	5	3%	0	0%
Small cell	6	1%	5	1%	1	1%	0	0%
Parosteal, dedifferentiated	19	2%	9	1%	7	5%	3	3%
Periosteal, high-grade	9	1%	6	1%	3	2%	0	0%
High-grade surface	10	1%	5	1%	4	3%	1	1%
Dedifferentiated LGCOS	4	0.5%	2	0.3%	2	1%	0	0%
Secondary	4	0.5%	0	0%	1	1%	3	3%
High-grade, NOS	150	17%	109	18%	23	15%	18	18%A

Abbreviations; LGCOS, low-grade central osteosarcoma; NOS, not otherwise specified. $p = 0.003$.

Supplementary Table 2. Multivariate analyses for disease-specific survival in accordance with age groups

	≤ 40 years				41–64 years				≥ 65 years			
	N	HR	95% CI	*p* value	N	HR	95% CI	*p* value	N	HR	95% CI	*p* value
Site												
Lower limb	506		Reference		100		Reference		58		Reference	
Upper limb	79	0.80	0.43–1.50	0.484	8	1.64	0.48–5.57	0.426	3	1.09	0.14–8.49	0.938
Trunk	30	2.45	1.32–4.58	0.005	46	1.32	0.64–2.72	0.457	41	1.26	0.59–2.70	0.558
Size												
≤ 8 cm	286		Reference		77		Reference		58		Reference	
> 8 cm	329	1.05	0.72–1.52	0.818	77	2.08	1.15–3.79	0.016	44	0.74	0.40–1.37	0.344
Surgery of primary tumor												
Yes	603		Reference		140		Reference		87		Reference	
No	12	1.64	0.49–5.49	0.422	14	1.65	0.65–4.21	0.294	15	3.02	1.03–8.87	0.045
Chemotherapy purpose												
No	9		Reference		16		Reference		58		Reference	
Adjuvant	563	5749.00	0–3.37E+46	0.863	121	1.16	0.39–3.51	0.789	35	1.04	0.53–2.05	0.908
Palliative	43	25329.61	0–1.48E+47	0.840	17	1.91	0.55–6.58	0.308	9	3.51	1.28–9.63	0.015
Radiotherapy purpose												
No	564		Reference		119		Reference		62		Reference	
Radical	7	4.54	0.26–4.54	0.918	4	1.44	0.19–11.11	0.726	5	3.42	1.09–10.72	0.035
Adjuvant	21	2.63	0.48–2.63	0.791	16	3.22	1.34–7.72	0.009	21	1.13	0.40–3.20	0.823
Palliative	23	9.90	3.38–9.90	<0.001	15	5.29	2.38–11.78	<0.001	14	1.34	0.52–3.48	0.549

Supplementary Table 3. Multivariate analyses for disease-specific survival in patients who underwent surgical treatments

	N	HR	95% CI	*p* value
Total	830			
Age				
≤ 40 years	603		Reference	
41–64 years	140	1.29	0.89–1.89	0.182
≥ 65 years	87	1.5	0.97–2.31	0.069
Site				
Lower limb	651		Reference	
Upper limb	90	0.91	0.53–1.56	0.732
Trunk	89	1.72	1.15–2.56	0.008
Size				
≤ 8 cm	405		Reference	
> 8 cm	425	1.13	0.85–1.51	0.392
Guideline-concordant treatment (perioperative chemotherapy + wide or radical resection)				
Yes	676		Reference	
No	154	1.54	1.09–2.18	0.016
Radiotherapy				
No	732		Reference	
Radical	16	1.74	0.80–3.80	0.163
Adjuvant	38	1.43	0.83–2.49	0.199
Palliative	44	4.98	3.36–7.38	<0.001

Supplementary Table 4. Multivariate analyses for disease-specific survival in patients who underwent guideline-concordant treatments

	N	HR	95% CI	*p* value
Total	676			
Age				
≤ 40 years	537		Reference	
41–64 years	107	1.56	1.02–2.39	0.042
≥ 65 years	32	1.97	1.10–3.52	0.023
Site				
Lower extremity	540		Reference	
Upper extremity	77	0.72	0.36–1.43	0.347
Trunk	59	2.24	1.41–3.56	<0.001
Size				
≤8 cm	322		Reference	
>8 cm	354	1.13	0.80–1.60	0.477
Radiotherapy				
No	618		Reference	
Radical	10	1.88	0.74–4.78	0.182
Adjuvant	21	1.94	0.97–3.88	0.062
Palliative	27	6.15	3.79–9.99	<0.001
